# The Role of Surface Treatment and Coupling Agents for Adhesion between Stainless Steel (SUS) and Polyamide (PA) of Heterojunction Bilayer Composites

**DOI:** 10.3390/polym16070896

**Published:** 2024-03-25

**Authors:** Hayeong Lee, Seung-In Song, Keon-Soo Jang

**Affiliations:** Department of Polymer Engineering, School of Chemical and Materials Engineering, The University of Suwon, Hwaseong 18323, Gyeonggi-do, Republic of Korea

**Keywords:** stainless steel (SUS), polyamide (PA), heterojunction bilayer composite, surface treatment, coupling agent, silane, lightweight, mechanical properties

## Abstract

The growing demand for lightweight and durable materials in industries, such as the automotive, aerospace, and electronics industries, has spurred the development of heterojunction bilayer composites, combining the structural integrity of metals with the versatility of polymers. This study addresses the critical interface between stainless steel (SUS) and polyamide 66 (PA66), focusing on the pivotal role of surface treatments and various silane coupling agents in enhancing the adhesion strength of heterojunction SUS/PA66 bilayer composites. Through systematic surface modifications—highlighted by scanning electron microscopy, atomic force microscopy, and contact angle analyses—the study assessed the impact of increasing the surface area, roughness, and energy of SUS. X-ray photoelectron spectroscopy evaluations confirmed the strategic selection of specific silane coupling agents. Although some coupling agents barely influenced the mechanics, notably, aminopropyl triethoxysilane (A1S) and 3-glycidyl oxypropyl trimethoxysilane (ES) significantly enhanced the mechanical properties of the heterojunction bilayer composites, evidenced by the improved lap shear strength, elongation at break, and toughness. These advancements were attributed to the interfacial interactions at the metal–polymer interface. This research underscored the significance of targeted surface treatment and the judicious selection of coupling agents in optimizing the interfacial adhesion and overall performance of metal–polymer composites, offering valuable insights for the fabrication of materials where reduced weight and enhanced durability are paramount.

## 1. Introduction

In recent years, heterojunction bilayer metal–polymer composite technology has emerged as a pivotal strategy to reduce the weight of products across the metal–polymer composite field [1,2,3]. These bilayer polymer–metal composites, which ingeniously meld the beneficial attributes of metals and polymers into a cohesive layered structure, are gaining traction across various industries, including automotive, aerospace, and electronics industries. Their growing popularity stems from their ability to combine the strength and durability of metals with the lightweight design and design versatility of polymers [4,5]. By integrating lightweight polymers with sturdy metals, the overall weight of the component can be significantly reduced, which is particularly beneficial in mobile applications like automotive and aerospace, where weight reduction is directly correlated with fuel efficiency and performance. The combination of heterojunction materials can also improve corrosion resistance and thermal stability with moderate mechanical properties depending on the specific metal and polymer used.

Robust adhesion between metal and polymer layers is paramount to obtaining heterojunction bilayer composites [6,7]. Adhesion can be enhanced by surface treatments and the development of coupling agents (compatibilizers) [8,9,10,11]. Stainless steel (SUS), known for its corrosion resistance, wear resistance, thermal resistance, workability, durability, and mechanical strength, plays a significant role across a wide array of industrial applications [12,13,14,15,16,17]. However, its surface characteristics can impede effective adhesion with polymers due to the inherent incompatibility between organic and inorganic materials [18,19]. To mitigate this phenomenon, various surface treatment techniques—including mechanical abrasion, chemical etching, plasma treatment, and laser modification—are utilized to enhance the adhesion capabilities of SUS; each method offers unique benefits to improve physical and interfacial interactions with polymers [20,21,22,23]. Mechanical abrasion is a common method where the surface is physically abraded to create a rougher texture, increasing the surface area and mechanical interlocking capabilities. The chemical treatment involves the use of acids or other chemicals to etch the surface, introducing rough surfaces and new functional groups that can form chemical or physical bonds with polymers. Plasma treatment is a sophisticated technique where the surface is exposed to plasma, altering its chemical composition and introducing polar groups that improve adhesion with polymers. The laser treatment uses focused laser beams to modify the surface texture and chemistry, offering precise control over the treatment.

Among the solutions to this challenge, silane-based coupling agents stand out for their ability to form a molecular bridge between inorganic and organic material surfaces, thereby substantially enhancing the interfacial bond strength [8,24,25]. The amphiphilic agents have a dual nature: one end (silane) of the molecule can bond with the metal, and the other end with the polymer, thus enhancing the bond strength between the two [26]. Each of these silane-based agents has unique properties and compatibility with different types of polymers [27]. The selection of these agents depends on the specific requirements of the polymer–metal interface, including the type of polymer, the desired properties of the final composite, and the conditions under which the composite will be used [28].

Polyamides, also named nylon, feature high mechanical strength, wear resistance, flexibility, chemical and thermal resistance, low friction coefficients, and electrically insulating properties and are lightweight [29,30,31,32,33,34,35]. Thus, it is extensively utilized in various applications, such as textiles, automotive applications, electronics, packaging, sports equipment, kitchen utensils, carpet fibers, and various household items, despite moisture absorption [36,37,38,39,40,41,42]. In particular, polyamides are commonly used in various automotive applications, such as brackets, housings, and structural elements [37].

**Figure 1:**
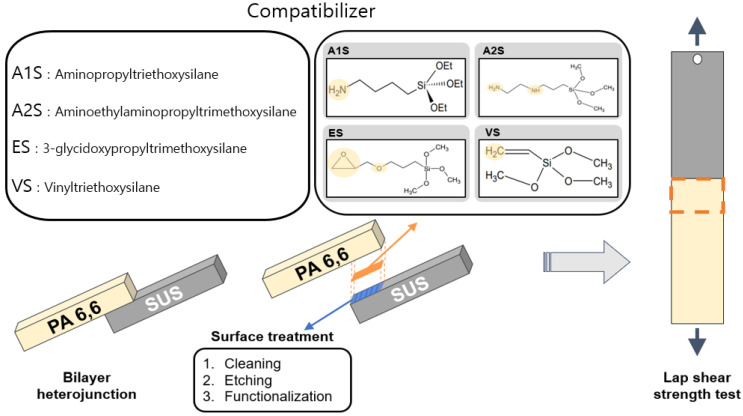
Combination of surface treatments for SUS and silane coupling agents (compatibilizer) for heterojunction bilayer SUS/PA66 composites. A1S: aminopropyl triethoxysilane; A2S: aminoethyl aminopropyl trimethoxysilane; ES: 3-glycid oxypropyl trimethoxysilane; VS: vinyl triethoxysilane.

This study aims to enhance the compatibility between SUS and PA66 in heterojunction bilayer SUS/PA66 composites through a strategic combination of surface treatments and the application of silane-based coupling agents, as shown in Figure 1. The surface treatments and silane-based coupling agents containing vinyl, epoxy, and amine (monoamine vs. diamine) functional groups were assessed through contact angle, morphology, element analysis, and thermal and mechanical properties. This research holds significant implications for various industrial applications where the synergy of metals and polymers is crucial for the performance and longevity of materials.

## 2. Experimental Section

### 2.1. Materials

**Figure 2:**
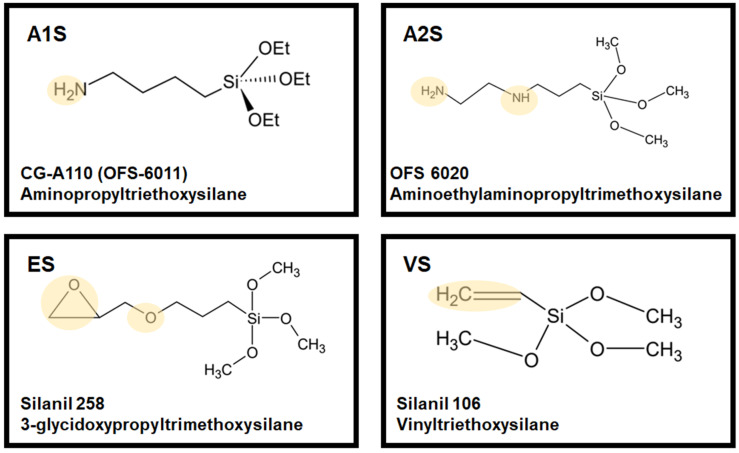
Structural formula of silane coupling agents.

Stainless steel 304 (SAE304 SUS) was obtained from Joontech Co. (Asan-si, Republic of Korea). Polyamide 66 (PA 66) was purchased from Ascend Performance Materials Co. (Seoul, Republic of Korea). The silane-based coupling agents, including aminopropyl triethoxysilane (A1S, OFS6011), aminoethyl aminopropyl trimethoxysilane (A2S, OFS6020), 3-glycid oxypropyl trimethoxysilane (ES, Silanil 258), and vinyl triethoxysilane (VS, Silanil 106), were acquired from BNOchem Co. (Cheongju-si, Republic of Korea). The structural formulas of the coupling agents are illustrated in Figure 2. Hydrochloric acid (HCl, 35–37%), copper(II) sulfate pentahydrate (CuSO_4_, 99%), ethanol (99.5%), and acetone were purchased from Samchun Pure Chemical Co. (Pyeongtaek-si, Republic of Korea). Sodium hydroxide (NaOH) was supplied from Duksan Chemical Co. (Ansan-si, Republic of Korea). Dihydrogen hexafluoro-zirconate (20% *w*/*w* in aqueous solution) was obtained from Alfa Aesar Co. (H_2_ZrF_6_, Ward Hill, MA, USA).

### 2.2. Surface Modifications of SUS Substrates

**Figure 3:**
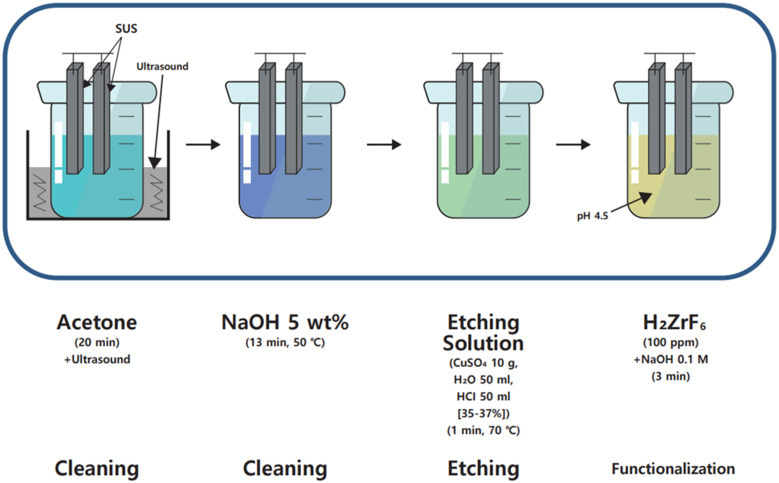
Surface treatment process of stainless-steel plates.

The surface treatment process of SUS is detailed in Figure 3. For thorough cleaning, the SUS was submerged in acetone, followed by ultrasonic cleaning for 20 min to remove any contaminants. An additional cleaning phase was performed, where the SUS specimens were immersed in a 5% (*w*/*v*) NaOH aqueous solution (5g NaOH + 100 mL deionized water) and subjected to cleaning at 50 °C for 13 min. Afterwards, to increase the surface area and energy of the SUS, it was immersed in the etching solution and etched for 1 min. The etching solution was formulated by combining 10 g of CuSO_4_, 50 mL of H_2_O, and 50 mL of HCl. The final step in the surface’s preparation involved immersing the SUS in a solution of H_2_ZrF_6_ (100 ppm) and NaOH (0.1 M) for 3 min to facilitate functionalization, thereby further improving the interfacial interactions between the SUS and the PA 66 polymer.

### 2.3. Compatibilization and Sample Fabrication for Lap Shear Strength Test

**Figure 4:**
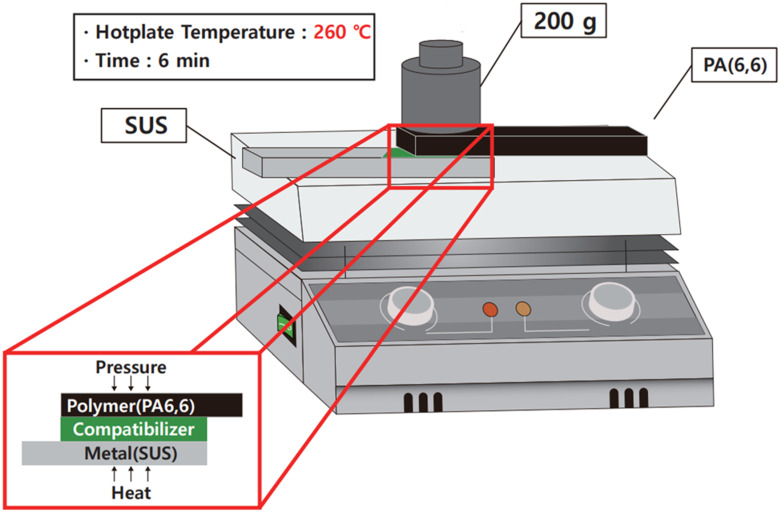
Fabrication of heterojunction bilayer composites for lap shear strength tests.

Following the surface treatment, 0.2 µL of each coupling agent was meticulously applied onto the SUS surfaces using a micropipette. The application was uniformly distributed across the bonding area with the aid of a brush. The solvent and moisture were eliminated on a hot plate at 90 °C for 3 min. For the bonding process, the surface-treated SUS was positioned on a hot plate preheated to 260 °C. The dimensions of SUS was 8.5 cm × 2.8 cm (23.8 cm^2^) whereas that of PA66 was prepared according to ISO 527-2 type 1A. A PA66 sheet with dimensions of 1.9 × 0.7 cm (1.33 cm^2^) was placed atop the SUS, as depicted in Figure 4. A weight of 200 g was applied over the designated bonding region to ensure uniform contact, and the assembly was subsequently heated for 6 min to facilitate the bonding process.

### 2.4. Characterization Techniques

#### 2.4.1. Scanning Electron Microscopy (SEM)

The surface morphologies of both untreated and surface-treated SUS specimens were observed using scanning electron microscopy (SEM; Apro, FEI Co., Hillsboro, OR, USA), applying an electron beam voltage of 10.0 kV and a magnification of ×2500. Samples for SEM measurements were obtained from lap shear strength tests. For SEM analysis, the samples were coated with a 5–10 nm thick gold layer using a sputter coater (Cressington 108 Auto Sputter Coater, Ted Pella Inc., Redding, CA, USA).

#### 2.4.2. Atomic Force Microscopy (AFM)

Atomic force microscopy (AFM, NX10, Park Systems Co., Suwon-si, Republic of Korea) was utilized to assess and quantify the SUS surfaces. Measurements were performed using a non-contact mode on specimens sized 1 mm × 1 mm, with scan settings of 10 μm × 10 μm and a rate of 1.0 Hz.

#### 2.4.3. X-ray Photoelectron Spectroscopy (XPS)

X-ray photoelectron spectroscopy (XPS, K-Alpha Plus, Thermo Fisher Scientific Co., Waltham, MA, USA) was utilized to confirm the application effectiveness of silane coupling agents on the SUS surfaces. Samples were cut into 1 mm × 1 mm dimensions via water jet cutting. Each sample underwent depth profiling. The samples without the silane coupling agent and those with ES were profiled for 20 s × 20 cycles, whereas those with A1S, A2S, and vs. were subjected to 40 s × 20 cycles for depth profiling.

#### 2.4.4. Contact Angle

The hydrophilicity of the SUS surfaces, both untreated and treated by various coupling agents, was determined by measuring the contact angle using a Digi-drop instrument (Phenix-MT(T), SEO Surface Electro Optics Co., Suwon-si, Republic of Korea). The contact angle was analyzed based on the morphology of images captured at the interfaces among air, droplet contours, and projections of the SUS surface. An average of five specimens was used for each measurement.

#### 2.4.5. Lap Shear Strength and Tensile Properties

The lap shear strength, elongation at break, and toughness of the bonded samples were obtained using a universal testing machine (UTM, LR10K Plus, Lloyd Instruments, AMETEK, Inc., Berwyn, PA, USA). The samples were measured at a constant speed of 4.8 mm/min at room temperature (22–24 °C). The average values for each sample were measured based on five specimens. Toughness was ascertained by integrating the area under the stress–strain curve.

## 3. Results and Discussion

This investigation focuses on enhancing interfacial adhesion through specific surface treatments of SUS and the strategic use of silane-based coupling agents comprising vinyl, epoxy, and amine (monoamine vs. diamine) functional groups. The surface modification of SUS is crucial for improving its compatibility with polyamide 66, thereby facilitating stronger bonding. The employment of silane coupling agents acts as a pivotal bridge, significantly improving the metal–polymer interface. The study meticulously evaluates the impact of these treatments through contact angle measurements, assessing surface wettability, and tensile testing to quantitatively analyze the adhesion strength between stainless steel and polyamide 66. Furthermore, thermal and mechanical analyses offer deeper insights into the adhesion’s resilience under varying conditions.

The contact angle measurements are conducted to gauge the wettability of the SUS surfaces, which is a direct indicator of the efficacy of surface modifications. Tensile tests were performed to quantitatively assess the adhesive strength between SUS and PA66. Moreover, thermal and mechanical analyses are incorporated to provide insights into the stability and durability of the adhesion under various conditions.

### 3.1. Morphology (SEM)

**Figure 5:**
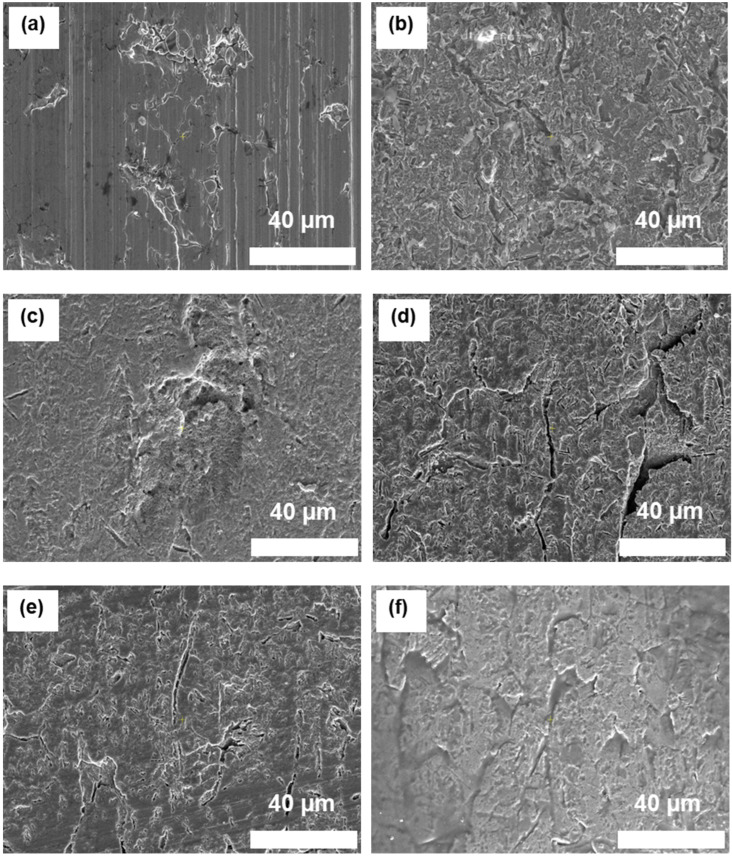
SEM images of surface-treated SUS surfaces with different silane coupling agents (×2500): (**a**) untreated SUS without coupling agents; (**b**) C+E+F-treated SUS without coupling agents; (**c**–**f**) C+E+F-treated SUS with coupling agents: (**c**) with A1S, (**d**) ES, (**e**) VS, and (**f**) A2S.

The impact of surface treatments and the application of silane-based coupling agents on surface morphology, roughness, and energy significantly influence the bonding strength of heterojunction bilayer composites. SEM imaging, as depicted in Figure 5 and Figure S1, illustrates the modifications in the surface morphology of SUS following treatment and subsequent coating with different coupling agents. The SEM images reveal that the surfaces of the SUS samples subjected to surface treatments (Figure 5b–f) displayed increased roughness compared with the untreated SUS sample (Figure 5a). This increased roughness is critical for enhancing mechanical interlocking at the interface. In addition, all surface-treated SUS specimens exhibited a uniform coating of the applied silane-based coupling agents (A1S, ES, A2S, and VS), indicating effective application on the prepared surfaces. Notably, the samples, which were surface-treated and then coated with A1S and A2S, presented a more substantial coating thickness, suggesting a denser layer of the coupling agent, which could potentially influence adhesion characteristics. This contrast in coating thickness between samples treated with A1S and A2S versus those with ES and vs. was also evident in the supplementary SEM images (Figure S1). This finding underscores the differential impact of the coupling agents on surface morphology and, by extension, on the adhesive properties of the heterojunction bilayer composites.

### 3.2. Morphology (AFM)

**Figure 6:**
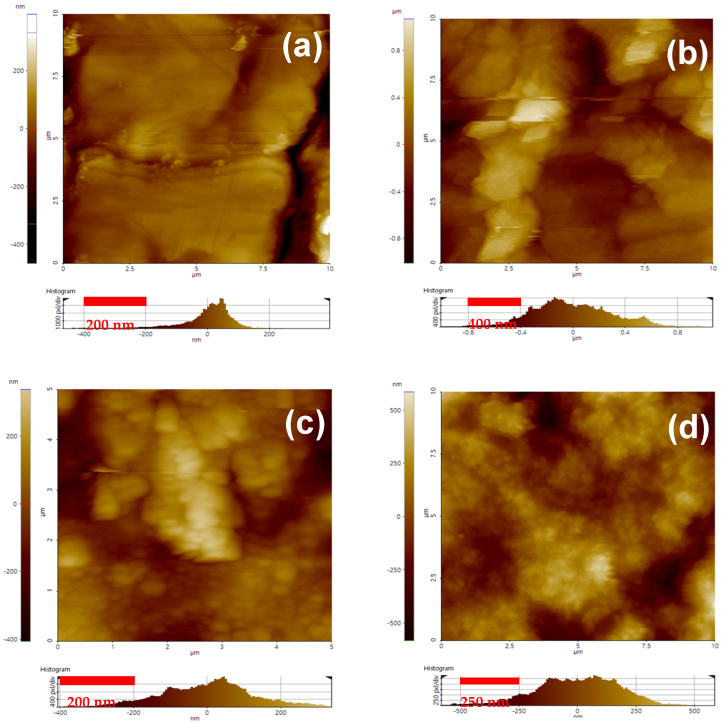

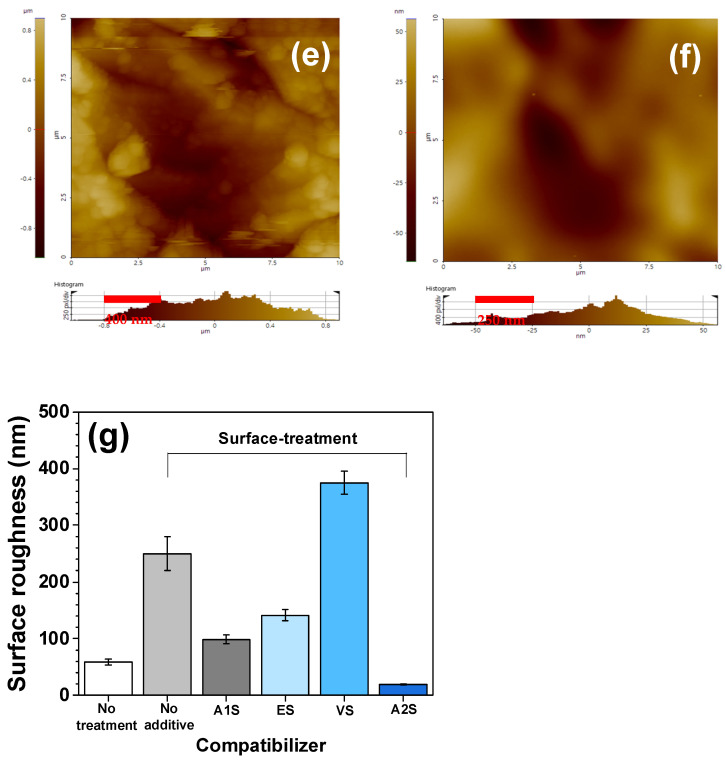
AFM 2S topography images with the height profile of SUS surfaces with different silane coupling agents: (**a**) untreated SUS without coupling agents; (**b**) C+E+F-treated SUS without coupling agents; (**c**–**f**) C+E+F-treated SUS with coupling agents: (**c**) with A1S, (**d**) ES, (**e**) vs., and (**f**) A2S; (**g**) average surface roughness of SUS surfaces with different silane coupling agents.

The strategic chemical modification of metal surfaces plays a crucial role in adjusting surface energy, roughness, and spatial dimensions, thereby significantly enhancing the adhesive strength of heterojunction bilayer composites. To precisely evaluate the alterations in surface roughness attributable to surface treatments, AFM was utilized. Figure 6 present the AFM images and the computed average surface roughness for SUS specimens treated with diverse silane coupling agents. It was observed that the average surface roughness of SUS notably increased following chemical surface treatment, indicating a successful modification. The subsequent application of silane coupling agents enabled the further quantification of surface roughness. The surface roughness of SUS reached its zenith with the application of VS, while it was minimized with A2S. Such disparities in surface roughness are indicative of the differential wetting behaviors, which are intricately linked to the intrinsic affinities of the coupling agents, as further explored in the ensuing discussion. Specifically, A2S and A1S, comprising amino functional groups, showed a pronounced affinity toward the SUS substrate, facilitating superior wetting and thus reducing surface roughness. By contrast, the inherently nonpolar vs. exhibited limited compatibility with the SUS substrate, resulting in challenging wetting conditions and a consequent increase in surface roughness.

### 3.3. Contact Angle

**Figure 7:**
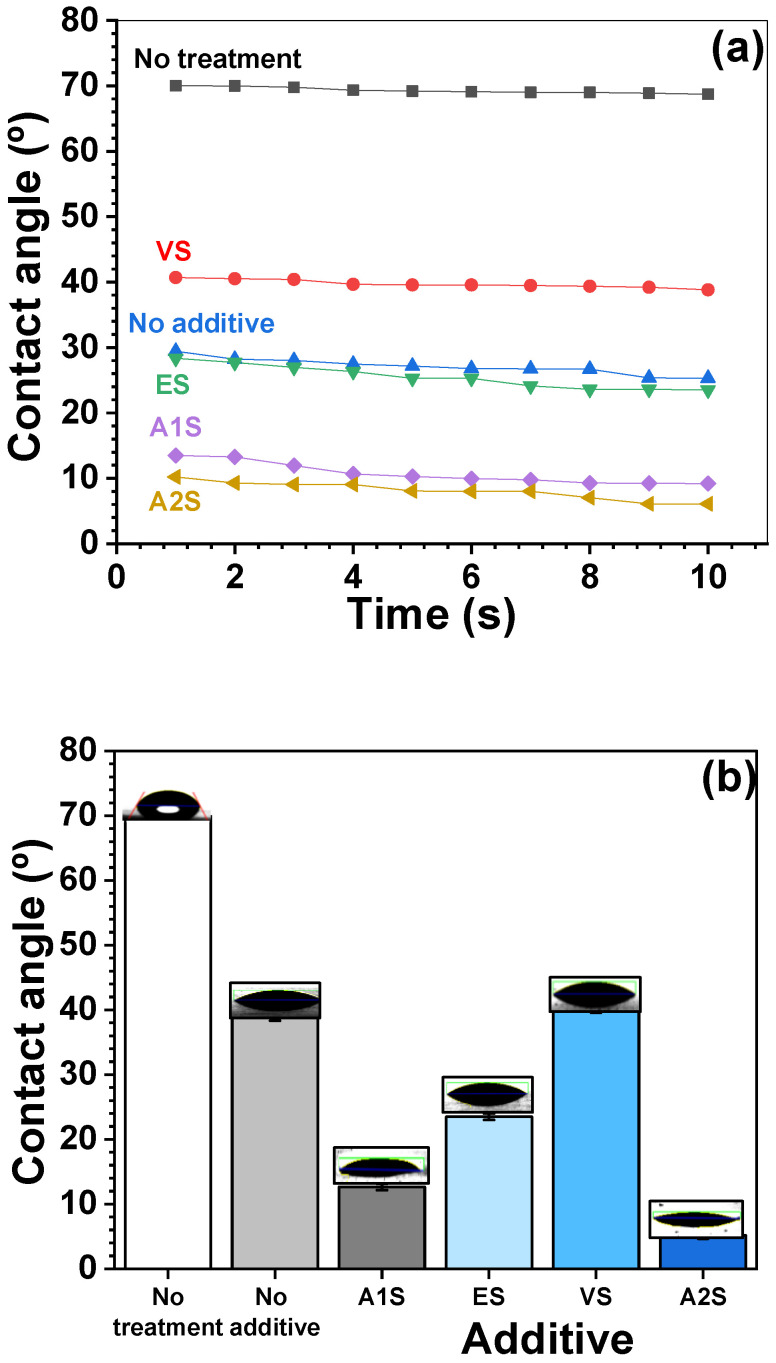
Contact angle of SUS coated with different silane coupling agents: (**a**) contact angle as a function of time; (**b**) contact angle using different additives.

The contact angles of untreated and surface-treated SUS with and without coupling agents were measured using deionized water (DIW), and the results are shown in Figure 7. The contact angle of untreated SUS was higher than the surface-treated SUS samples with and without coupling agents. This increased wettability was attributed to the increase in surface roughness, area, and energy caused by the surface treatment. Among the surface-treated SUS samples using a coupling agent, the largest contact angle was measured for the surface-treated SUS with VS, characterized by its non-polar functional group, whereas the smallest contact angle was measured for the surface-treated SUS with A2S, which contains two polar amino groups. This variation is likely due to the interaction between the polar amino groups of A2S and the polar DIW [43]. The presence of polar functional groups on the surface-treated SUS significantly influenced the contact angle and wettability.

### 3.4. X-ray Photoelectron Spectroscopy (XPS)

**Figure 8:**
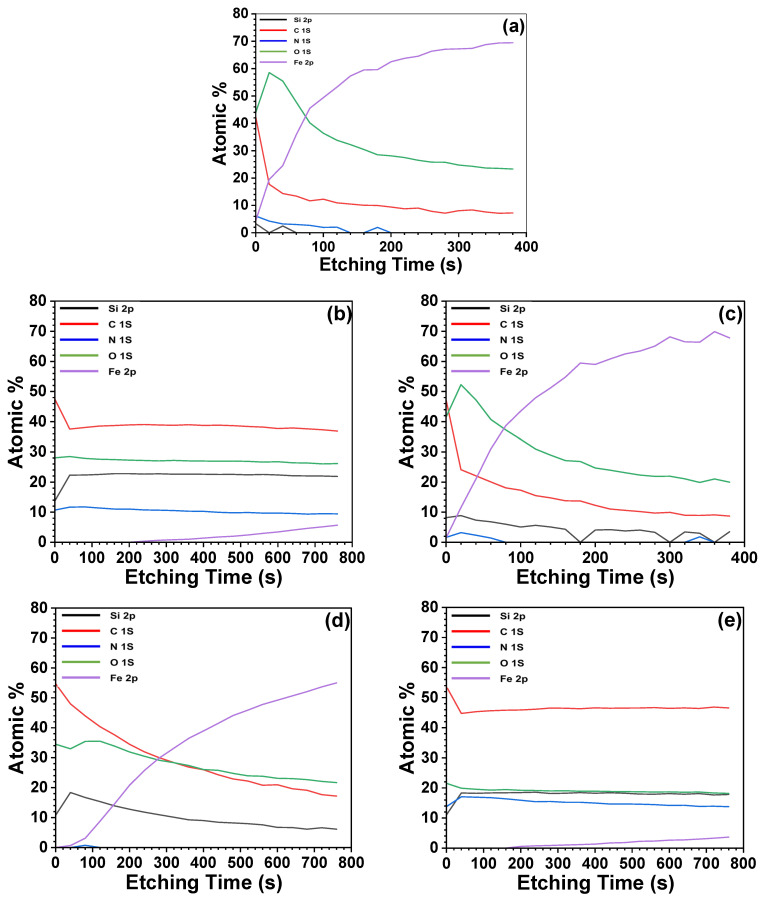
XPS depth profiling of C+E+F-treated SUS surfaces with different silane coupling agents: (**a**) none, (**b**) with A1S, (**c**) ES, (**d**) VS, and (**e**) A2S.

To ascertain the effectiveness of the silane coupling agent application on the SUS surface, XPS measurements, including depth profiling, were conducted. Depth profiling is a method of using an ion beam to sequentially etch and analyze the surface or contamination layers [44]. Figure 8 shows a notable increase in the atomic percentage of Fe 2p, along with a decrease in C 1s, O 1s, and Fe 2p percentages as a function of etching time. Samples coated with A1S and A2S revealed the presence of N 1s, indicative of amino functional groups, with A1S and A2S incorporating one and two amino functional groups, respectively. The initial detection of N 1s in other samples is likely attributable to organic contaminants on the surface. A reduction in the N 1s atomic percentage with prolonged etching confirms the thorough application of the silane coupling agent. Moreover, the atomic percentage of Fe 2p in A1S- and A2S-coated samples barely increased when compared with other specimens. This finding corroborates the SEM observations in Figure 5; that is, A1S and A2S formed a denser coating, and this is likely due to their self-protecting thickness resulting from the aggregation of amide groups. The elevated atomic percentages of O 1s in uncoated, and ES-coated SUS samples can be attributed to the metal oxide layer and oxirane groups, respectively. Furthermore, the atomic percentage of Fe 2p in all specimens gradually increased with increasing etching time, reflecting the iron content of the SUS substrate.

### 3.5. Mechanical Properties (UTM)

**Figure 9:**
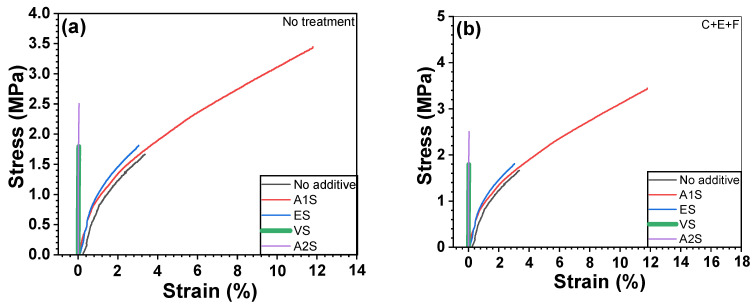

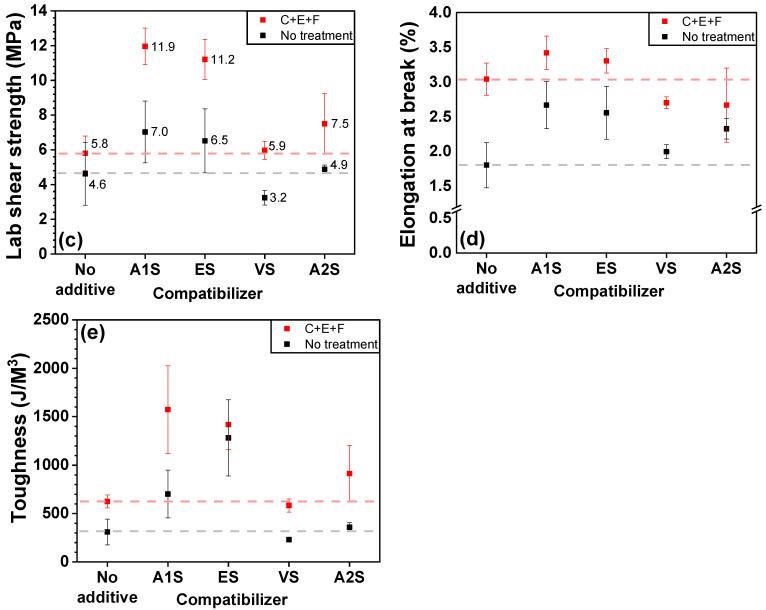
Mechanical properties of PA66-based heterojunction bilayer composites containing untreated and surface-treated SUS coated with different coupling agents: (**a**) stress–strain curves of untreated SUS, (**b**) stress–strain curves, (**c**) lap shear strength, (**d**) elongation at break, and (**e**) toughness.

In this research, the role of silane coupling agents in enhancing the interfacial interactions between metal and polymer in heterojunction bilayer composites was evaluated [26]. The investigation into the mechanical characteristics of heterojunction SUS/PA66 bilayer composites, both with and without the application of coupling agents, is depicted in Figure 9. The lap shear strength, elongation at break, and toughness of these composites were quantitatively assessed using a UTM. The assessment included composites that received no pretreatment as well as those subjected to a comprehensive surface treatment regimen consisting of cleaning, etching, and functionalization (C+E+F). The specimens benefiting from surface treatment exhibited superior mechanical properties compared to their untreated counterparts, a phenomenon attributable to the enhanced surface roughness and area, corroborated by SEM and AFM analyses. The use of A1S and ES on the SUS surfaces markedly enhanced the mechanical properties of the heterojunction bilayer composites, including lap shear strength, elongation at break, and toughness, compared to the samples devoid of coupling agents. This enhancement was particularly pronounced with A1S, suggesting that hydrogen bonding between the amino groups of A1S and the amide functions of PA66 significantly contribute to the observed mechanical property improvements. In contrast, the application of A2S, despite possessing two amino groups, rarely yielded significant mechanical enhancements. This condition is potentially attributed to the detrimental effects of amino group agglomeration. Similarly, the positive impact of ES on mechanical properties likely stems from interactions between the amide groups of PA66 and the terminal epoxide groups of ES. Conversely, VS, characterized by its nonpolar structure, exhibited limited interaction with the PA 66 polymer, resulting in negligible improvements in mechanical characteristics. The trends observed in elongation at break and toughness closely mirrored those of lap shear strength. This suggests that A1S and ES played the coupling role at the interface between SUS and PA66. The results of the contact angle and the mechanical properties were similar to each other. However, in the case of A2S, although the contact angle was the lowest, it did not yield good mechanical properties. This finding appears to be because A2S has high hydrophilicity, which is beneficial, but the internal hydrogen bonding among amino groups within A2S acts as a negative role, barely increasing the mechanical properties.

### 3.6. Morphology of Fractured SUS and PA66 Surfaces (SEM and Camera Images)

**Figure 10:**
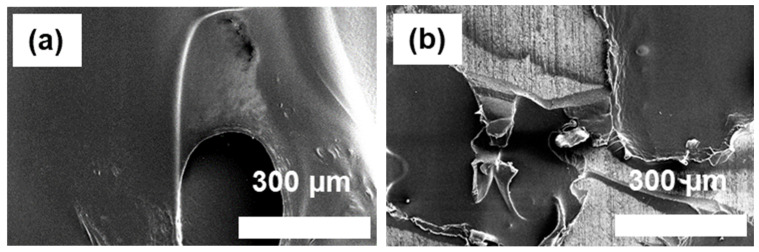

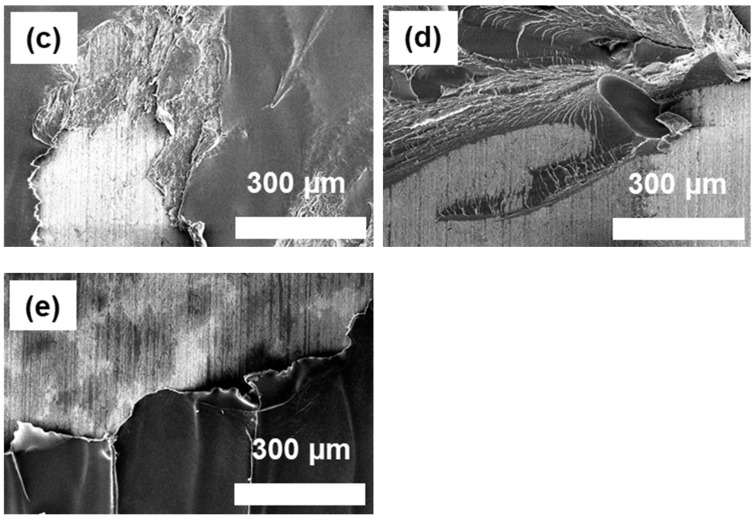
SEM images of fractured SUS surfaces with different silane coupling agents (×250) after lap shear tests: (**a**) C+E+F-treated SUS without coupling agents; (**b**–**e**) C+E+F-treated SUS with coupling agents: (**b**) with A1S, (**c**) ES, (**d**) VS, and (**e**) A2S.

**Figure 11:**
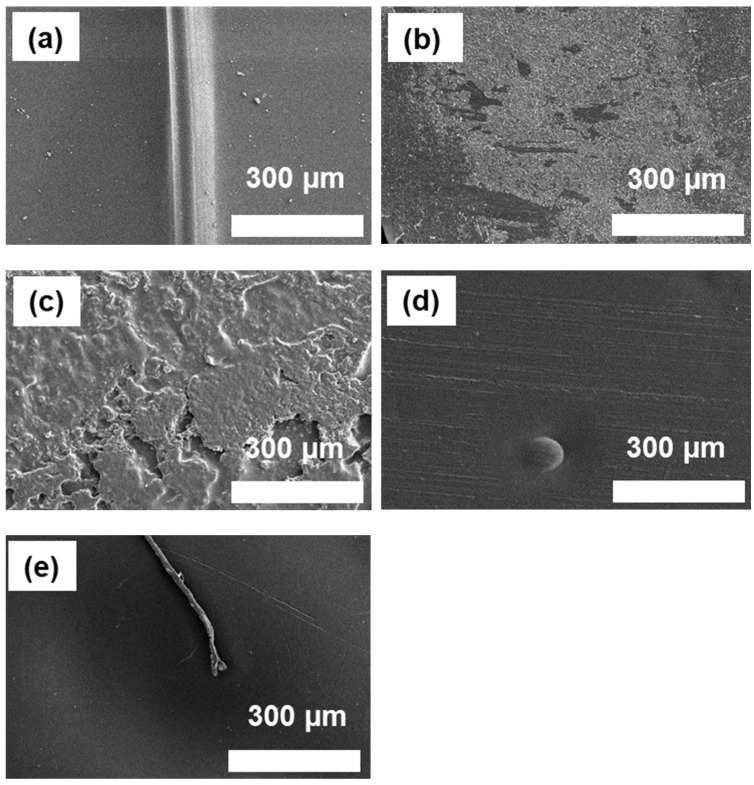
SEM images of fractured PA66 surfaces with different silane coupling agents (×250) after lap shear tests: (**a**) C+E+F-treated SUS without coupling agents; (**b**–**e**) C+E+F-treated SUS with coupling agents: (**b**) with A1S, (**c**) ES, (**d**) VS, and (**e**) A2S.

The morphologies of fractured SUS and PA66 surfaces subsequent to the lap shear tests are depicted in Figure 10, Figure 11 and Figures S3–S5. Fractured surfaces treated with A1S and ES exhibited distinct features, including less smooth interfaces, more pronounced color/contrast gradients, and a higher prevalence of pore-like structures compared to those treated with VS, A2S, and those without coupling agents. These observed variations in color and morphology across the samples can be attributed to the establishment of robust interfacial interactions between SUS and PA66. The trends observed in SEM analysis mirror those identified in mechanical testing, whereby interfaces characterized by low interfacial interactions displayed smoother surfaces and diminished color gradients.

### 3.7. Summary of Results 

Table 1 summarizes all the results of this study. In terms of surface roughness, vs. exhibited the highest value, while A2S had the lowest. This is because VS, being a nonpolar group silane, forms bonds via van der Waals forces with minimal intermolecular interaction, resulting in the highest measured surface roughness by AFM. On the other hand, A2S, containing amino groups and exhibiting hydrophilicity, showed significantly higher intermolecular interaction, leading to the lowest measured surface roughness. Regarding the contact angle, the untreated sample (without treatment) showed the highest angle, while A2S, possessing two amino groups with strong hydrophilic properties, exhibited the lowest contact angle when measured with deionized water. The lap shear strength, elongation at break, and toughness were measured. Samples treated with A2S, which contained two amino groups, showed relatively lower mechanical properties probably due to the self-stacking/entanglement caused by interactions among the amino groups of A2S rather than interactions between amino groups and PA66. Among the treated samples, A1S had the highest values, whereas among the untreated samples, the sample treated with VS, which contains nonpolar groups, had the lowest values.

## 4. Conclusions

This study extensively explored the efficacy of surface treatments and the application of silane coupling agents in enhancing the mechanical properties of heterojunction bilayer composites, specifically SUS/PA66 composites. Through meticulous experimentation, including SEM, AFM, and XPS analyses and mechanical testing, we have elucidated the significant impact of chemical modification on the surface characteristics of SUS and its subsequent effect on composite performance. The application of surface treatments to SUS increased surface roughness and energy, thereby improving wettability, as evidenced by decreased contact angles in surface-treated and coupling agent-coated SUS samples. The enhanced wettability indicates an improved interfacial interaction between the metal and polymer layers, a critical factor for the performance of heterojunction bilayer composites. Among the evaluated silane coupling agents (A1S, A2S, ES, and VS), A1S and ES were particularly effective in enhancing the mechanical properties of the composites, including lap shear strength, elongation at break, and toughness due to their polar groups, which are similar characteristics to those in PA66. This enhancement was attributed to the formation of hydrogen bonds between the amino groups of A1S or the epoxide groups of ES with the amide moieties of PA66, facilitating strong interfacial bonding. Conversely, A2S and vs. showed minimal to no improvement in mechanical properties, which can be ascribed to the agglomeration effect in the case of A2S and the low affinity of nonpolar vs. with PA 66. The depth profiling and surface analysis through XPS further confirmed the successful application of coupling agents, revealing the presence of specific functional groups that contributed to the observed enhancements in adhesion and mechanical properties. In summary, this study exhibited the critical role of surface treatment and the strategic selection of silane coupling agents in optimizing the interface between metal and polymer layers in heterojunction bilayer composites. Our findings provide a solid foundation for future studies to tailor the interfacial properties for specific industrial applications, particularly where the synergy of metals and polymers is crucial for material performance and longevity. Future work will focus on exploring other coupling agents and treatment methods to broaden the application scope of such composites in advanced engineering fields:

✓ We extensively investigated the effectiveness of surface treatments and silane coupling agents in improving the mechanical properties of heterojunction bilayer composites, specifically SUS/PA66 composites.
✓ We utilized SEM, AFM, and XPS analyses and mechanical testing to elucidate the significant impact of chemical modification on SUS surface characteristics and subsequent composite performance.
✓ Surface treatments increased surface roughness and energy, enhancing wettability as evidenced by reduced contact angles in surface-treated and coupling agent-coated SUS samples and indicating improved interfacial interaction for composite performance.
✓ A1S and ES demonstrated notable effectiveness in enhancing composite mechanical properties, and this is attributed to their polar groups akin to PA66, facilitating strong interfacial bonding through interfacial interactions with amide moieties.
✓ Depth profiling and XPS surface analysis confirmed successful coupling agent applications, highlighting the specific functional groups contributing to the enhanced adhesion and mechanical properties and offering valuable insights for tailoring interfacial properties in future composite design for diverse industrial applications.

## Figures and Tables

**Table 1:** Overall results with/without surface treatments and with different compatibilizers in this study where the red and blue colors indicate the highest and lowest values, respectively.

	Pristine	No Additive	A1S	ES	VS	A2S
Surface roughness	58.7 nm	250 nm	98.6 nm	141.6 nm	375 nm	19.4 nm
Contact angle	70.01°	38.8°	12.6°	23.5°	40.1°	5.1°
Lap shear strength with treatment	-	5.8 MPa	11.9 MPa	11.2 MPa	5.9 MPa	7.5 MPa
Lap shear strength without treatment	-	4.6 MPa	7.0 MPa	6.5 MPa	3.2 MPa	4.9 MPa
Elongation at break with treatment	-	3.0%	3.4%	3.3%	2.7%	2.6%
Elongation at break without treatment	-	1.8%	2.6%	2.5%	1.9%	2.3%
Toughness with treatment	-	626 J/M^3^	1570 J/M^3^	1418 J/M^3^	584 J/M^3^	914 J/M^3^
Toughness without treatment	-	310 J/M^3^	702 J/M^3^	1282 J/M^3^	230 J/M^3^	359 J/M^3^

## Data Availability

The raw data supporting the conclusions of this article will be made available by the authors on request.

## Supplementary Materials

The following supporting information can be downloaded at: https://www.mdpi.com/article/10.3390/polym16070896/s1. Figure S1. Fabrication of heterojunction bilayer composites for lap shear strength tests. Figure S2. SEM images of untreated SUS surfaces with different silane coupling agents (×2500): (a) A1S, (b) ES, (c) A2S, and (d) VS. Figure S3. SEM images of fracture-surface SUS with different silane coupling agents (×2500) after lap shear tests: (a) C+E+F-treated SUS without coupling agent, (b–e) C+E+F-treated SUS with coupling agent: (b) with A1S, (c) ES, (d) VS, and (e) A2S. Figure S4. SEM images of fractured PA66 surfaces with different silane coupling agents (×2500) after lap shear tests: (a) C+E+F-treated SUS without coupling agent, (b–e) C+E+F-treated SUS with coupling agent: (b) with A1S, (c) ES, (d) VS, and (e) A2S. Figure S5. SEM images of fractured SUS and PA66 surfaces with different silane coupling agents: (a) C+E+F-treated SUS without coupling agent, (b–e) C+E+F-treated SUS with coupling agent: (b) with A1S, (c) ES, (d) VS, and (e) A2S. Left and right images indicate SUS and PA66, respectively, after lap shear tests.
