# Supplementary material for: The Role of Surface Treatment and Coupling Agents for Adhesion between Stainless Steel (SUS) and Polyamide (PA) of Heterojunction Bilayer Composites
Source: Polymers (Basel). 2024 Mar 25;16(7):896. doi: 10.3390/polym16070896 (PMC11013711; doi:10.3390/polym16070896)
Supplement: Supplementary file 1 [file polymers-16-00896-s001.zip › polymers-2915252-supplementary.pdf]

Supplementary information

Role of Surface Treatment and Coupling Agent for Adhesion between Stainless Steel (SUS) and Polyamide (PA) of Heterojunction Bilayer Composites

Hayeong Lee[1], *Seung-In Song*[1], *and Keon-Soo Jang**

Department of Polymer Engineering, School of Chemical and Materials Engineering, The University of Suwon, Hwaseong, Gyeonggi-do,18323, Republic of Korea

[1]: These authors (H. Lee and S. Song) contributed equally: Co-1st authors

CORRESPONDING AUTHOR FOOTNOTE *To whom correspondence should be addressed. K.-S. Jang: ksjang@suwon.ac.kr

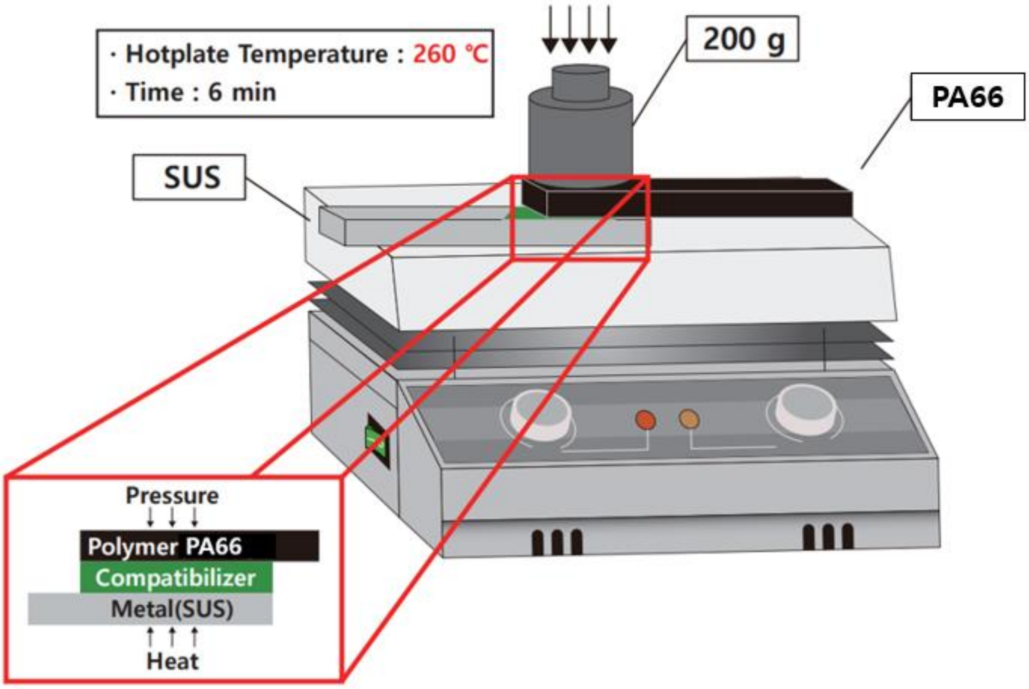

Figure S1. Fabrication of heterojunction bilayer composites for lap shear strength tests.

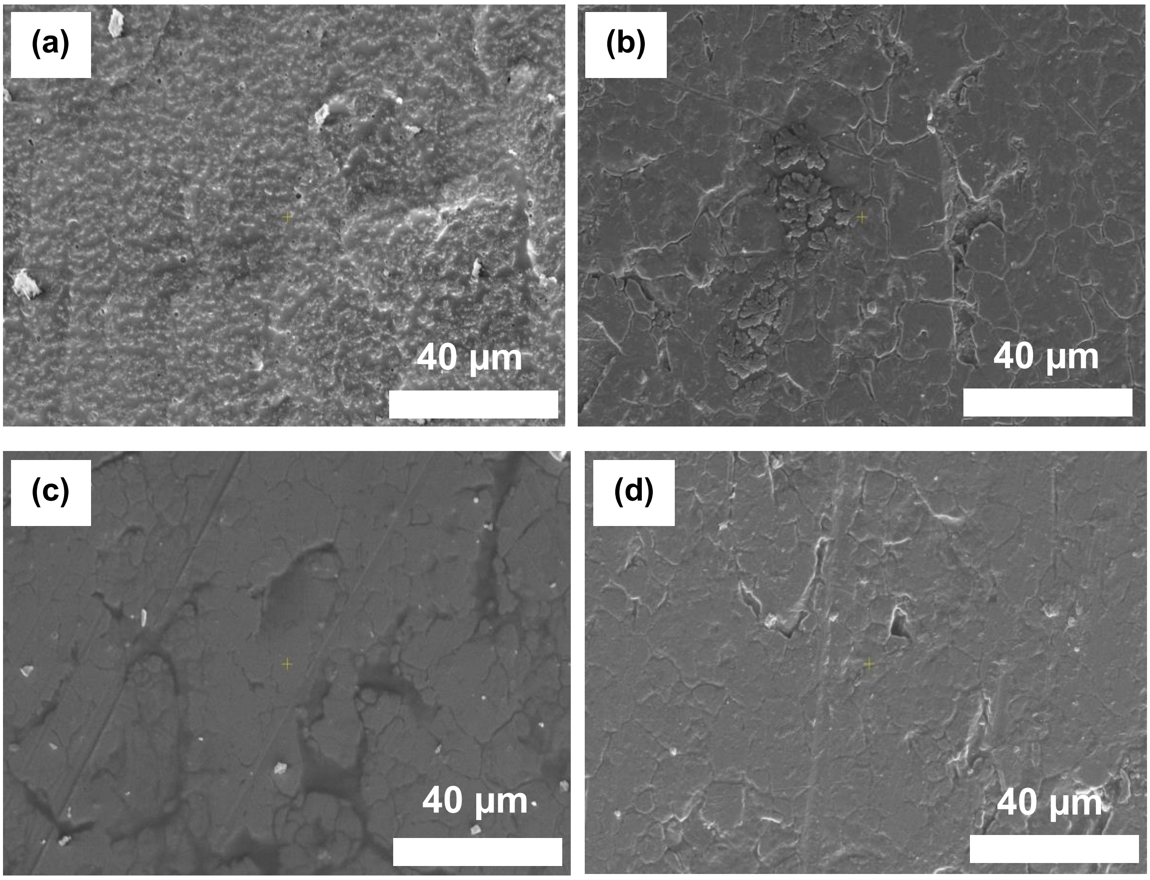

Figure S2. SEM images of untreated SUS surfaces with different silane coupling agents (×2,500): (a) A1S, (b) ES, (c) A2S, and (d) VS.

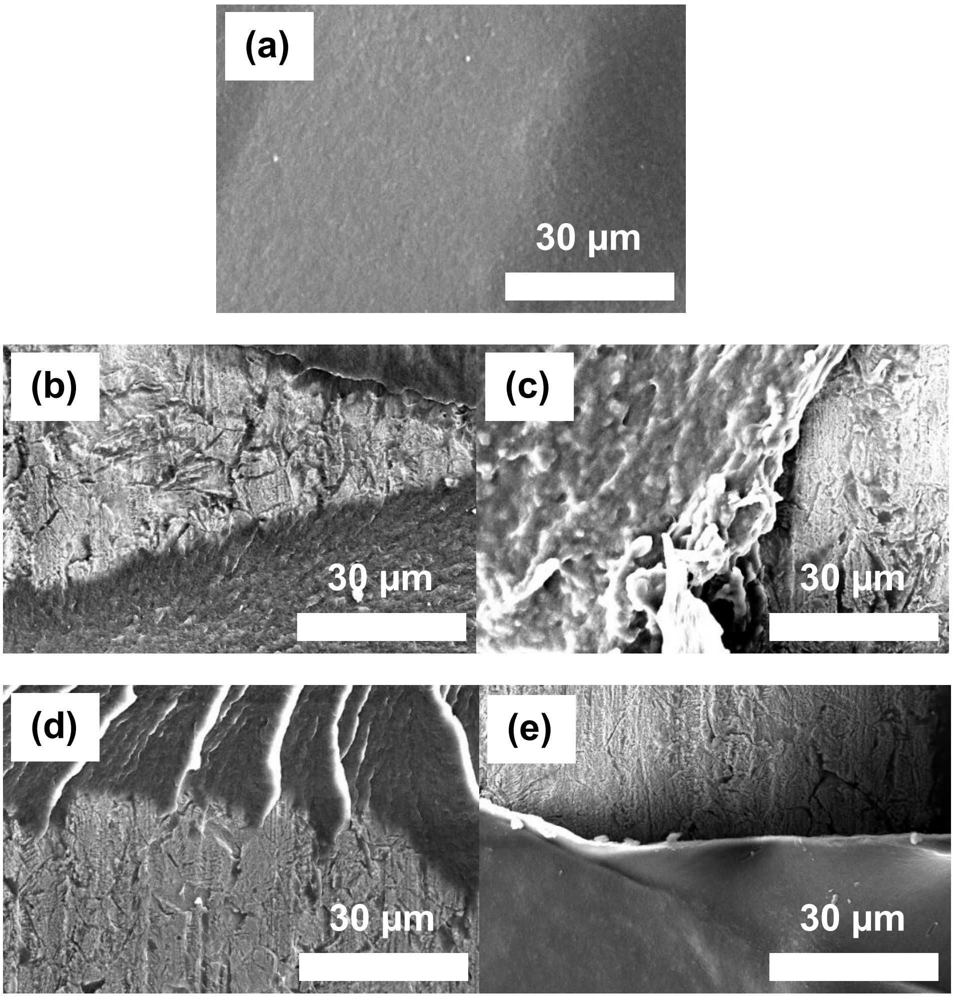

Figure S3. SEM images of fracture-surface SUS with different silane coupling agents (×2,500) after lap shear tests: (a) C+E+F-treated SUS without coupling agent, (b–e) C+E+F-treated SUS with coupling agent: (b) with A1S, (c) ES, (d) VS, and (e) A2S.

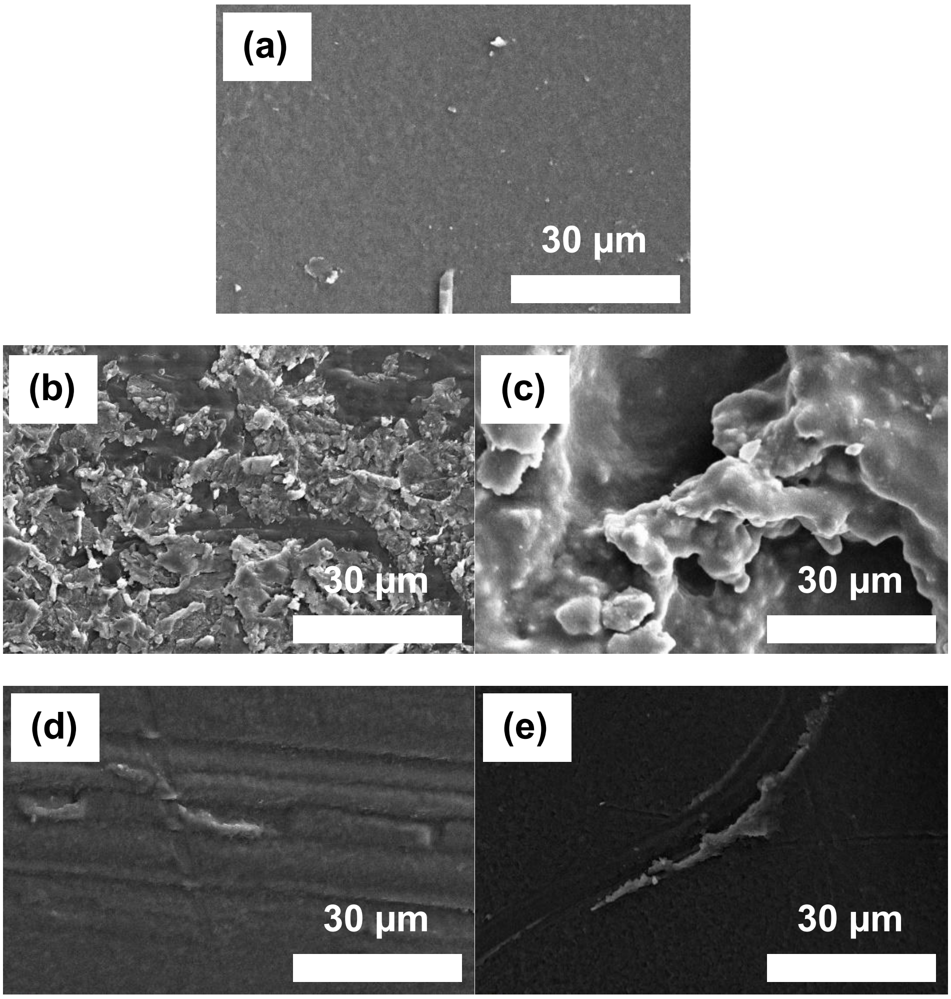

Figure S4. SEM images of fractured PA66 surfaces with different silane coupling agents (×2,500) after lap shear tests: (a) C+E+F-treated SUS without coupling agent, (b–e) C+E+F-treated SUS with coupling agent: (b) with A1S, (c) ES, (d) VS, and (e) A2S.

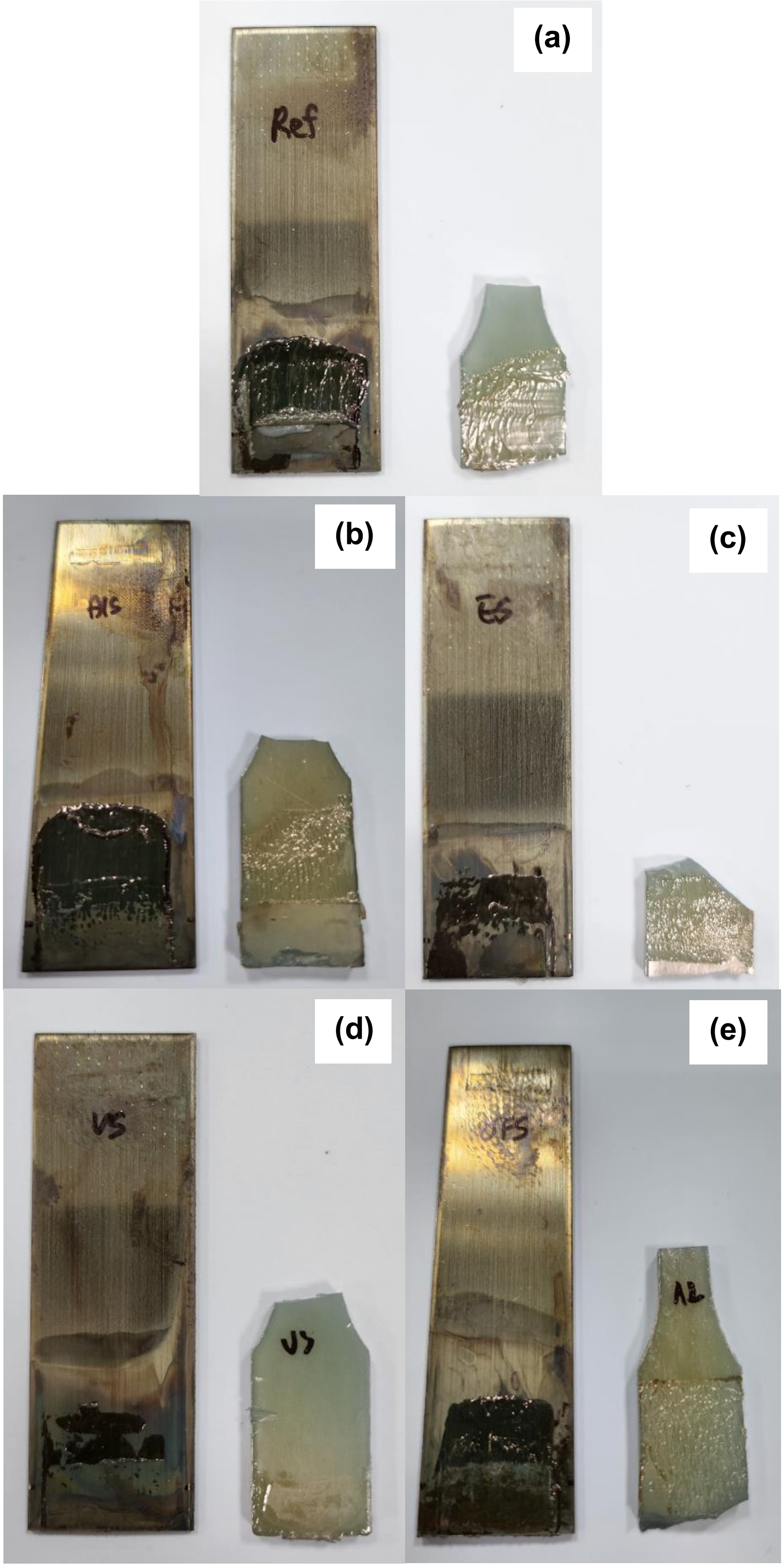

Figure S5. SEM images of fractured SUS and PA66 surfaces with different silane coupling agents: (a) C+E+F-treated SUS without coupling agent, (b–e) C+E+F-treated SUS with coupling agent: (b) with A1S, (c) ES, (d) VS, and (e) A2S. Left and right images indicate SUS and PA66, respectively, after lap shear tests.
